# Trend Analysis of Malaria Occurrence in Wolaita Zone, Southern Ethiopia: Retrospective Cross-Sectional Study

**DOI:** 10.1155/2015/123682

**Published:** 2015-12-07

**Authors:** Deresse Legesse, Yusuf Haji, Solomon Abreha

**Affiliations:** College of Health Sciences and Medicine, Wolaita Sodo University, P.O. Box 138, Wolaita Sodo, Ethiopia

## Abstract

*Background.* Malaria is a major public health problem in Ethiopia. The trend of malaria occurrence remains unknown in the study area. This study is aimed at determining the last five years' trend of malaria occurrence from 2008/09 to 2012/13 in Wolaita Zone, Southern Ethiopia.* Methods.* A health facility-based retrospective study was conducted in Wolaita Zone from March to August, 2014. Five years' laboratory confirmed malaria record review was made from six health centers.* Result.* A total of 105,755 laboratory confirmed malaria cases were reported, with total slide positivity rate of 33.27% and mean annual occurrence of 21,151 cases. Malaria occurred with a fluctuating trend in the study area, with its peak occurring at the year 2011/12. Overall, no remarkable decline in the total laboratory confirmed malaria was observed in the last five years.* P. falciparum* was the predominantly reported species, accounting for 75,929 (71.80%) of cases. The highest slide positivity rate was observed in the age group of 5–14 years (40.5%) followed by 1–4 years (35.5%). Two malaria peak seasons occurred: one from September to December and the other from April to June.* Conclusion.* No remarkable decline in laboratory confirmed malaria in the last five years was observed.

## 1. Introduction

Malaria is endemic in Ethiopia with varying intensity of transmission [1]. The country is also one of the most malaria epidemic-prone countries in Africa with rates of morbidity and mortality increasing dramatically during epidemics [2]. Despite efforts and considerable progress in malaria intervention over the past decades, the disease still remains to be a major public health concern in Ethiopia [3]. According to records from the Ethiopian Federal Ministry of Health, 75% of the country is malarious with about 68% of the total population living in areas at risk of malaria. That is, more than 50 million people are at risk of malaria, with an estimated 4-5 million cases and 70,000 deaths occurring every year [4, 5]. Besides the individual suffering caused by the disease, malaria poses immense socioeconomic burden in the country [1, 6].

In Ethiopia,* Plasmodium falciparum* and* Plasmodium *vivax are the two predominant malaria parasites, are distributed all over the country, and are responsible for 60% and 40% of malaria cases, respectively, and there are two peak seasonal transmissions of malaria occurring during the months of September to December and March to May [2].

Malaria is an entirely preventable and treatable disease, provided the currently recommended interventions are properly implemented. These include vector control through the use of long-lasting insecticide-treated nets (LLINs), indoor residual spraying (IRS), and, in some specific settings, larval control, chemoprevention for the most vulnerable populations, particularly pregnant women and infants, confirmation of malaria diagnosis through microscopy or rapid diagnostic tests (RDTs) for every suspected case, and timely treatment with appropriate antimalarial medicines. According to World Health Organization (WHO), the strategic approaches to malaria control come within two major domains: prevention and case management. Accordingly, World Health Organization recommends universal access to and utilization at household level of long-lasting insecticide-treated nets (LLINs) and indoor residual spraying (IRS) as the most powerful and effective strategies of vector control, to rapidly control malaria transmission, hence reducing the burden of malaria morbidity and mortality [7].

In 2011, the World Health Assembly, Roll Back Malaria Partnership, set the objectives: to reduce malaria cases by 75% and malaria deaths to near zero from 2000 levels by 2015. And these objectives of mortality and morbidity reduction are linked to targets of achieving universal access to case management and universal access to and utilization of malaria prevention measures [7]. According to World Malaria Report 2014, globally, malaria cases decreased from 227 million in 2000 to 198 million in 2013 and the incidence is projected to fall by 35% globally and by 40% in the WHO African Region by 2015. In sub-Saharan Africa, the proportion of the population protected by at least one vector control method has increased in recent years; it reached 48% in 2013, and the number scaled up in 2014. The prevalence of malaria parasite infection, including both symptomatic and asymptomatic infections, has decreased significantly with average infection prevalence in children aged 2–10 years falling from 26% in 2000 to 14% in 2013, a relative decline of 46% [8].

Prevention and control activities of malaria in Ethiopia are implemented according to the National Strategic Plan (NSP) for Malaria Control and Prevention that operates in line with the WHO recommendations. The National Strategic Plan for Malaria Control and Prevention in Ethiopia, NSP 2011–2015, is aimed at strengthening and scale-up of malaria control interventions through prompt and effective diagnosis and treatment, case management through roll-out of the highly efficacious antimalaria drugs, Artemisinin-based combination therapies (ACTs), and selective vector control with special emphasis to scaling up of LLINs coverage and ensuring its utilization at household level, and targeted and timely application of IRS of households with insecticide and environmental management. The strategic plan has set goals to achieve malaria elimination in areas with historically low malaria transmission and near zero malaria deaths in all the remaining parts of the country by 2015. To attain these goals, it has set out the following specific targets: 100% of households in malarious areas own one LLIN per sleeping space, at least 80% of people at risk of malaria use LLINs, IRS coverage is increased and maintained to 90% of households in IRS-targeted areas, 100% have access to effective and affordable malaria treatment [2, 9].

As outlined in the NSP 2011–2015, Ethiopia has a target of 100% access to effective and affordable malaria treatment. This requires improving diagnosis of malaria cases using microscopy or using multispecies rapid diagnostic tests (RDTs) and providing prompt and effective malaria case management at all health facilities in the country. Malaria diagnosis consists of a patient's clinical assessment, microscopic examination of blood slides, and use of multispecies RDT in accordance with the level of the health facility. Microscopic examination of blood slides performed in accordance with the WHO standard operating procedure (SOP) is regarded in the National Malaria Diagnosis and Treatment Guidelines as the national standard of malaria diagnosis in health centers and hospitals across the country, whereas multispecies RDTs are the main diagnostic tool at the health post level. ACTs are the first-line drug for treatment of uncomplicated* P. falciparum* malaria [2, 9].

The national Malaria Indicator Survey (MIS) 2011 that was conducted following the scale-up of malaria control interventions in Ethiopia showed achievements in coverage of some malaria control interventions and malaria indicators between 2007 and 2011 [10]. Malaria showed a decline in Ethiopia over the last ten years as a result of high coverage of key malaria control interventions. This is attributable to the introduction of ACTs, use of rapid diagnostic tests (RDTs) at the peripheral health facilities, wide-scale distribution of long-lasting insecticidal nets (LLINs), and high coverage of sprayed households through targeted indoor residual spraying [1]. On the other hand, recent studies conducted across different parts of the country reveal that, despite the decrease in malaria morbidity and mortality attributed to the introduction of the current malaria control strategies, malaria still remains to be a major health problem of the country with unstable occurrences and fluctuating trends. According to a study on prevalence of malaria from blood smears examination from a hospital in Northwest Ethiopia, malaria was observed in almost every month of the year with overall slide positive rate of 17%, with slight decrease from 2007 to 2012 [11]. Another facility-based malaria trend analysis in Gondar, Northwest Ethiopia, revealed that the burden of malaria was high in the study area with slide positivity rate of 39.6%, the disease occurring throughout the year with fluctuating trends [3].

## 2. Significance of the Study

It is obvious that the global malaria status is the sum total of each country status, and in turn the country status is the sum total of its regional and local situations at each level. Accurate assessments of the levels and time trends in malaria burden are crucial for the assessment of progress towards goals and planning national health services and focusing future efforts [12, 13].

Despite the aforementioned studies at different parts of the country, malaria situation with regard to its trend, seasonal patterns, and distribution, particularly the case of the last five years' period, remains unknown in the study area. Therefore, this study was aimed at determining the trend of malaria occurrence in Wolaita zone over the last five years' period from 2008/09 to 2012/13, or September 2001–August 2005, according to Ethiopian Calendar (E.C.). The study provides scientific evidence that would be an important data base of local, national, and global relevance in advancing current knowledge on malaria situation. It is also useful to policy makers and program planners at each level for assessing progress and focusing future efforts while providing evidence-driven public health action in preventing and controlling malaria incidence.

## 3. Objectives

### 3.1. General Objective

The study is generally aimed at determining the trend of malaria prevalence in the last five years' period from 2008/09 to 2012/13 in Wolaita zone, Southern Ethiopia.

### 3.2. Specific Objectives

The specific objectives are as follows:

- To describe malaria occurrence in Wolaita zone.
- To determine the trend of malaria occurrence for the last five years' period from 2008/09 to 2012/13 in the area.

## 4. Methods and Materials

### 4.1. Study Area and Period

The study was conducted in Wolaita zone, one of the 14 zones in Southern Nations Nationalities and Peoples Region (SNNPR), Ethiopia. The zone has total area of 4512 square kilometers, administratively divided into 12 districts (locally termed “woredas”) and 3 town administrations with total population of nearly 1.7 million. It is composed of 3 agroecological zones: 9% highland (“Dega”), 56% midland temperate (“Woinadega”), and 35% lowland (“Kolla”), with altitude ranging from below 1000 meters at the foot of Omo river valley to 2,950 meters above sea level. The average temperature varies from 15°C to 31°C, and the annual rainfall has characteristic monthly variation, with peak rainy seasons usually observed during March through May (autumn), locally termed as “belg,” and July through September (summer to partially spring), locally termed as “kremt” to partially “tsedey.” In the zone are currently 1 governmental and 2 nongovernmental hospitals and 11 health centers aged 5 years or above as of establishment. The study was carried out in six of these health centers (Badessa, Bale, Gasuba, Humbo, Shanto, and Sodo health centers). Each of these health centers serves various catchment populations depending on their location or proximity to towns. Accordingly, catchment population of 57,721 is served by Sodo health center, whereas Humbo health center serves a total population of 40,604. Each of Gasuba, Badessa, Shanto, and Bale health centers serves catchment population of 36,652, 28,000, 24,560, and 22,417, respectively. The study was conducted from March to August 2014.

### 4.2. Study Design

A health facility-based retrospective cross-sectional study was conducted.

### 4.3. Source Population

The total catchment population of Wolaita zone was the source population of the study.

### 4.4. Study Population

Those individuals who were blood film tested within the last five years (2008/09–2012/13) in the selected health facilities of Wolaita zone were included in the study population.

### 4.5. Sampling Technique

As most of uncomplicated malaria cases usually present to primary health facility level, and to maintain consistency of data across all the catchment areas and woredas for representation of the zone, we purposively considered only health centers. There are 11 health centers in the zone aged 5 years or above as of establishment, of which we randomly selected six health centers (50%) by lottery method. Accordingly, Badessa, Bale, Gasuba, Humbo, Shanto, and Sodo health centers were included in the study.

### 4.6. Data Collection Procedure

Daily malaria report data collection form including name of health institution, year, month, date, total number of BFs seen, and sex, age, and residence with each positive species type (*P. falciparum*,* P. vivax*, and mixed infection) has been developed for data collection. Data was collected by medical laboratory technicians or nurses with diploma level. Review of routine laboratory confirmed malaria case report was made from the selected health facilities. Daily malaria data with the above relevant variables was extracted from the institutional malaria registration book of the last five years' period from 2008/09 to 2012/13 (2001–2005 E.C.). Analysis of the six health centers' routine daily based laboratory confirmed case reports combined across dates, months, and years was made to determine malaria occurrence.

### 4.7. Data Processing and Analysis

Data was entered by date. That is, individual daily data including health center name; year; date; total BF seen; number of P. f. positive with sex, age category, and resident; number of P. v. positive with sex, age category, and resident; and number of mixed infections with sex, age category, and resident has been entered into Epi Info 3.5.3. Data cleaning and analysis were performed by Excel worksheet. Annual malaria occurrence was determined by health centers,* Plasmodium* species, sex, and age groups. Line graphs are used to depict the overall as well as specific trends of malaria prevalence in the last five years.

### 4.8. Data Quality Management

The study used cases that were confirmed at health centers by the nationally used standard of malaria diagnosis and laboratory examination of blood films and extracted these cases from the national malaria registration book of the health facilities. One-day training was given for data collectors on data extraction. The overall process of data collection was followed up by the principal investigator and field supervisors. For instance, a sample of completed data collection form was randomly selected and checked daily by the principal investigator for accuracy, incompleteness, and inconsistency. All completed data was once again rechecked manually just before entry. Finally, the data was cleaned for analysis.

### 4.9. Ethical Considerations

Official letter was sought from Wolaita Sodo University Research and Community Service Directorate and permission to undertake the study (review record) was obtained from every relevant authority at all levels; Wolaita Zone Health Department; and respective woreda health offices as well as administrative bodies of respective health facilities.

## 5. Result

### 5.1. Overall Malaria Burden

A five-year retrospective study was conducted in six health centers of Wolaita zone. A total of 317,867 blood films were seen in all the six health centers within the five years' period from 2008/9 to 2012/3 (2001–2005 E.C.). Among these, a total of 105,755 laboratory confirmed malaria cases were reported from all the six health centers, with total slide positivity rate (SPR) of 33.27% and mean annual prevalence of 21,151 cases.

### 5.2. Malaria Occurrence by Health Center, Plasmodium Species, Sex, and Age Groups

Among the six health centers considered, relatively largest number of prevalent cases was reported from Gasuba health center, with 31,435 (29.72%) of all cases. Of the total confirmed malaria cases, the predominantly reported species was* P. falciparum*, accounting for 75,929 (71.80%) of the total occurrence, followed by* P. vivax* and mix of both species infection with* P. falciparum* and* P. vivax*, each constituting 25,329 (23.95%) and 4,497 (4.25%), respectively. The disease affected both males and females almost equally, accounting for 53,662 (50.74%) and 52,093 (49.26%) of cases, respectively.

**Figure 1:**
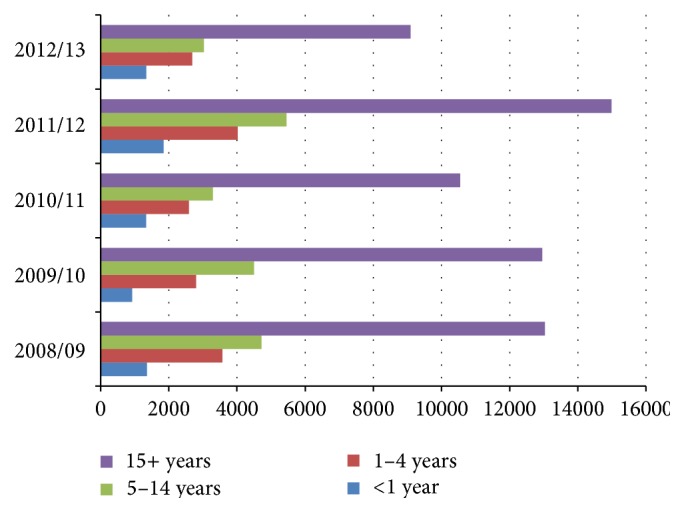
Total malaria occurrence by age groups, Wolaita zone, Southern Ethiopia, 2008/09–2012/13.

Majority of reported cases were in the age group of ≥15 years, overall accounting for 62,182 (58.80%) of cases, and this is also consistent across each of the prevalent species as well as across each of the five years (Figure 1). Regarding malaria incidence rate per each specific age group, the highest slide positivity rate was observed in the age group of 5–14 years followed by 1–4 years. Out of 51,842 total blood films examined in the age group of 5–14 years, 20,991 were tested positive with age-specific slide positivity rate of 40.5%, whereas out of 48,862 total blood films examined in the age group of 1–4 years, 17,339 were tested positive with slide positivity rate of 35.5%. The slide positivity rate was observed to be relatively low in the age group of <1 year (Table 1).

### 5.3. Trend Analyses

#### 5.3.1. Overall Five Years' Trend of Malaria Occurrence

**Figure 2:**
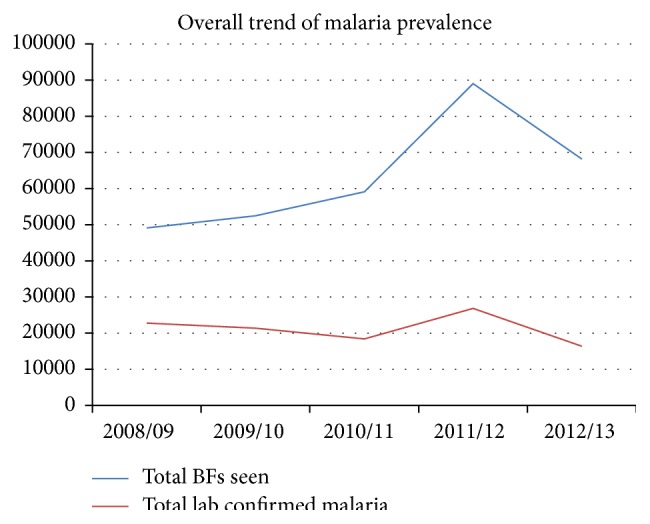
Five years' trend of laboratory confirmed malaria prevalence in Wolaita zone, Southern Ethiopia, 2008/09–2012/13.

Overall, malaria showed a fluctuating trend in the zone during the last five years' period from 2008/9 to 2012/13. The prevalence had shown slight fall from 2008/09 to 2010/11 for three consecutive years. Then, from 2010/11 to 2011/12, it had shown a sharp rise for the next one year until it reached its peak at 2011/12. Pick malaria case was reported during the year 2011/12 (2004 E.C), and then the prevalence had shown a sharp fall till the recent year, 2012/13. Overall, however, no considerable reduction in laboratory confirmed malaria prevalence was observed for the last five years' period (Figure 2).

#### 5.3.2. Trend of Malaria Occurrence by Woreda Health Centers

**Figure 3:**
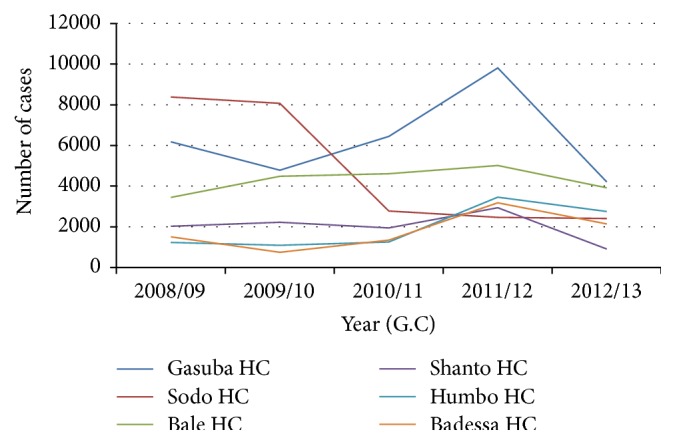
Trend of laboratory confirmed malaria occurrence by woreda health centers, Wolaita zone, Southern Ethiopia, 2008/09–2012/13.

The highest malaria occurrence was observed in Sodo health center consecutively from 2008/9 till about half of the year 2010/11 where trend shift was made by Gasuba health center, where onwards the highest prevalence was took over by Gasuba health center consistently for all the years later. In Sodo health center, significant decline in malaria prevalence was observed, with consistent decline from 2009/10 onwards to the year 2012/13, whereas in Gasuba health center, it rose from 2009/10 to 2011/12 but showed a sharp fall for next one year, maintaining relatively highest prevalence. Low and increasing prevalence was observed in Badessa and Humbo health centers, although it had shown relative decline for the recent one year, whereas relatively stable prevalence was observed in Bale health center. Malaria prevalence reached peak in the year 2011/12 (2004 E.C.) consistently across all the six health centers (Figure 3).

#### 5.3.3. Patterns and Trends by Plasmodium Species

**Figure 4:**
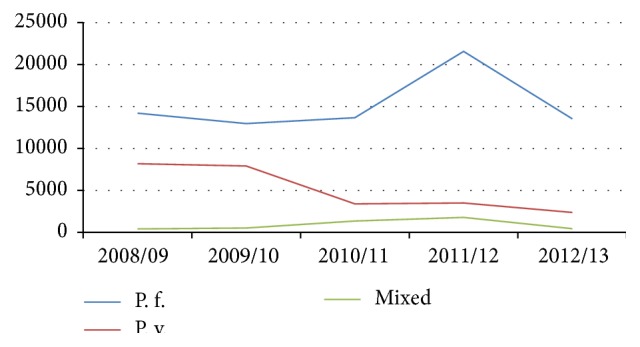
Malaria trend by* Plasmodium* species, Wolaita zone, Southern Ethiopia, 2008/09–2012/13.

Of the total confirmed malaria cases, the predominantly reported species was* P. falciparum*, accounting for 75,929 (71.80%) of the overall prevalence, followed by* P. vivax* and mixed infection with* P. falciparum* and* P. vivax*, each constituting 25,329 (23.95%) and 4,497 (4.25%), respectively. Overall, this distribution was maintained consistent throughout all the five years' period with all peaks at the year 2011/12. However, there were fluctuations in respective trends over the years.* P. falciparum*, between 2009/10 and 2011/12, and mixed infection, between 2008/09 and 2011/12, showed relative increase, although both showed sharp decline for the recent one year. But* P. vivax* continued to show consistent decline across the five years' period (Figure 4).

#### 5.3.4. Seasonal Variation of Malaria Occurrence

**Figure 5:**
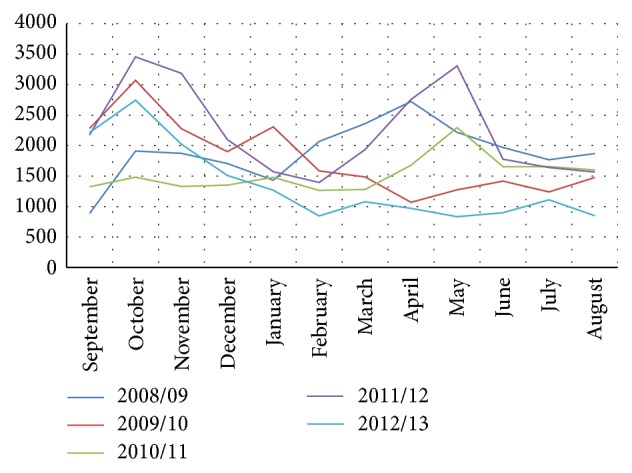
Seasonal variation of malaria prevalence in Wolaita zone, Southern Ethiopia, 2008/09–2012/13.

Although malaria occurred in all months, the prevalence had shown fluctuating trend across months in all of the study years. Two peak seasons occurred in the years 2008/09, 2010/11, and 2011/12, with the first peak between September and November and the second between April and June, but one peak between September and November (only the first peak) occurred in the years 2009/10 and 2012/13. Overall, two malaria peak seasons occurred; the first peaks occurred between September and November (spring) and the second peaks occurred between April and June (autumn through summer). Both peaks were the highest and seasonal variation was significant in the year 2011/12 (2004 E.C.) (Figure 5).

## 6. Discussion

In this study, a total of 105,755 laboratory confirmed malaria cases were reported within the last five years' period from 2008/9 to 2013/13 (2001–2005 E.C.), with total slide positivity rate of 33.27% and mean annual occurrence of 21,151 cases. Although this finding is in coincidence with the world estimate including African regions [13] and it is consistent with studies from different parts of the country, it could be an important indicator for existence of malaria burden in the study area, which seems to need due attention with regard to malaria intervention during this critical period of the national and global striving towards reducing malaria burden in the year 2015. Both the average annual occurrence and the total slide positivity rate in our study are lower as compared to the study in Kola Diba, Northern Ethiopia, in which the mean annual occurrence was about 2,347 cases and the total slide positivity rate was 39.6% [3], but it is higher when compared to the finding from Metema Hospital, Northern Ethiopia, in which the slide positivity rate was 17% [11]. These discrepancies might be attributed to the geographical setup, variations in the study setting, or other factors.

Among the six health centers considered in the zone, overall, relatively largest number of prevalent cases was reported from Gasuba health center, with the trend increasing consecutively for the years 2009/10 to 2011/12, although it declined for the recent one year. This variation might be because of the presence of high malaria breeding sites attributed to relative agroecological differences that might be making some parts of the area such as this woreda relatively malarious in the zone. It might also result from differences in habits of routine documenting and reporting of cases and relative differences in the catchment population served by the respective health centers, and the increasing trend observed for two-year periods might be related to inconsistencies with efforts of malaria intervention activities.

According to* Plasmodium* species distribution in Ethiopia,* P. falciparum* and* P. vivax* are the two predominant malaria parasites occurring in the country accounting for 60% and 40% of malaria cases, respectively [2]. In our study, the predominantly reported species was* P. falciparum* with 75,929 (71.80%) of the overall occurrence, followed by* P. vivax* and mixed infection of both species. This is in agreement with findings revealed from various studies [5, 14, 15]. The predominance with* Plasmodium* species was maintained consistent across all of the five years despite relative fluctuations in trends.* P. falciparum* and mixed species showed fluctuations over the years, with relative increase until the usual peak at 2011/12, while* P. vivax* malaria continued to show consistent decline across the five years' period. This finding might be natural phenomena occurring under normal conditions.

According to reports and most of studies conducted abroad and here in Ethiopia, males are highly affected by malaria than females which is perceived to be attributed to the fact that males are usually engaged in outdoor activities that put them at greater risk of contracting the disease [5, 6]. In our study, the distribution is nearly equal with only slight variations. This might be due to differences in the study area, relative differences with regard to gender roles, or other factors. It might also be an important indicative that temporal changes might be avoiding gender differences. Regarding distribution of malaria prevalence by age groups, majority of reported cases were in the age group of ≥15 years consistently across years and across each of the prevalent species, with 62,182 (58.80%) of the overall prevalence. The same findings were observed in studies across different regions of the country [3, 5, 11]. The predominance by the adult population could be normally related to the relative proportion of the adult population and is an important indication that the disease affected largely the productive age group in the study area, with an implication for due attention for protecting lives of the country's development force in line with achieving development goals. The highest slide positivity rate was observed among the age group of 5–14 years (40.5%) followed by 1–4 years (35.5%). This finding is in agreement with the finding from Metema Hospital, Northwest Ethiopia, in which the age groups of 5–14 years were highly affected by malaria infection, followed by 15–29 years [11], but it slightly varies from the study of Kola Diba, North Gondar, in which the age groups of 15–44 years were more affected, with a prevalence rate of 50.1%, followed by 5–14 and 1–4 years of age groups with the prevalence rate of 20% and 19.6%, respectively [3]. The current finding is an important implication that these age groups need due attention with regard to malaria intervention, particularly prevention and control measures performed at household level.

Malaria showed a fluctuating trend in the zone during the last five years' period from 2008/9 to 2012/13, with its peak at 2011/12. The fluctuating occurrence of malaria is observed in various similar studies [3, 14]. Consistent occurrence of the peak prevalence in the year 2011/12 (2004 E.C.) observed across all the woreda health centers of the zone, except Sodo health center, was found to be questioning in the study area. It might be explained possibly by remarkable loosening in malaria intervention measures in woredas all over the zone or an epidemic that could have occurred in this particular year. Decline in the prevalence observed in the recent year might be attributed to relative scale-up of malaria interventions in the study area. However, further evidence on sustainability of the trend to the years later is important to ascertain such attributes.

The largest occurrence relatively predominated by Sodo health center for more than one year's period from 2008/09 seems to be largely due to the larger catchment population served by the health center. The health center located in the town could also be accompanied by high population density, relative flow of individuals to the town from surrounding local villages, temporary residents, and casual visitors. However, the consistent decline observed from 2009/10 onwards till the year 2012/13 could be attributed to strengthened integration of malaria intervention activities in the town, which is still requiring further information for the years later to ascertain sustainability of the decline to the most recent years. In spite of its low occurrence, malaria prevalence was observed to be consistently ever increasing throughout majority of the study periods in Badessa and Humbo health centers, while relatively stable occurrence was observed in Bale and Shanto health centers. These variations observed among woredas could be related to relative variations in agroecological characteristics, relative differences in malaria intervention performance, documenting, and reporting and are suggestive of the importance of due attentions.

In this study, annual malaria occurrence was observed to have seasonal variation in all of the study years. Two malaria peak seasons occurred: the first peaks occurred from September to December (spring, the main rainy season in Ethiopia) and the second peaks occurred between April and June (autumn and partially summer). This finding slightly deviates from the report based on 12 years' data analysis in India, in which peak malaria seasons were observed from mid-June to September [16]; and it also slightly varied from the pattern reported in South Korea, where July and August were found to be the most prevalent months, with one peak occurring between June and October [17], but it coincides with the pattern in Sudan, where malaria cases are high during July to November, strongly influenced by average monthly rainfall during this rainy season [18]. The seasonality observed in the current study is in agreement with studies in different parts of Ethiopia, all of which reveal seasonal variation on incidence of malaria in the country, while most reveal annually occurring continuous bimodal transmission episodes [3, 6, 11, 14]. The peak seasonal occurrence observed in this study is found to be coinciding with the relatively high annual rainy seasons ecologically characterizing the study area. It might be related to high transmission periods during these seasons, which might be explained by ecological characteristics and environmental factors facilitating vector breeding, temporal changes in herd immunity, vector characteristics, socioeconomic conditions, factors related to health care services, changes in life style, or other factors.

In this study, the level of malaria occurrence might be underestimated as secondary data might be incomplete and cases counted are only those self-reported. However, as these kinds of errors are not systematic in nature, we believe the observed trend is not therefore affected. It was based on data from six health centers, that is, more than 50% of all health centers in the zone that stayed five years or above of its establishment. This composition we believe is great in making the finding strong in providing credible evidence about malaria in the study area, and it provides practical grounds for sound comparisons.

## 7. Conclusion

Malaria remains as a public health problem in the study area with relatively high slide positivity rate. It occurred with a fluctuating trend during the last five years' period, with its peak occurring during 2011/12. Across the years, no consistent fall is observed in the trend except for the recent one year; overall, there was no remarkable decline in laboratory confirmed malaria in the last five years' period. This could be an important indicative that the area is hardly on the track, with implications for need of great emphasis to malaria intervention in line with achieving the global as well as the national objectives of reducing malaria burden in 2015.* P. falciparum* was the predominantly reported species. The highest slide positivity rate was observed in the age group of 5–14 years followed by 1–4 years. Seasonality was observed in occurrence of malaria, with two malaria peak seasons occurring annually.

## 8. Recommendations

Based on our study findings, we strongly forward the following recommendations.

Strong emphasis should be given to malaria prevention and control at zonal as well as woreda levels, with an effort to avert incidence of malaria in the study area; performance plans and achievements should be regularly and strictly reviewed and evaluated at each level.

Malaria intervention measures should be strengthened and scaled up in the study area with comprehensively integrated activities implemented in line with the national malaria guidelines, striving towards the right track of the national and global objectives of reducing malaria burden in the year 2015.

As it is revealed by studies that use of LLINs can reduce both malaria transmission and its mortality by a considerable proportion, great emphasis should be given to LLIN distribution and ensure its utilization at every household in the study area, along with other vector control measures such as environmental management.

Public awareness creation could be important through strong health extension activities integrated with the community.

Special attentions need to be considered for some woredas such as Badessa and Humbo woredas, where malaria prevalence was observed to be consistently ever increasing throughout majority of the study periods.

Seasonal variations should be well recognized with special focus given to the annual peak seasons (months), to prevent high malaria transmissions and potential outbreaks.

Further studies should be conducted to determine or forecast potential epidemic patterns in the study area.

## Figures and Tables

**Table 1:** Distribution of slide positivity rate among different age groups in Wolaita zone, Southern Ethiopia, 2008/09–2012/13.

Age in years	Total BFs seen	Total BFs positive	
		Frequency	Percentage
<1 year	23310	6811	29.2%
1–4 years	48862	17339	35.5%
5–14 years	51842	20991	40.5%
≥15 years	193853	60614	31.3%
Total	317,867	105,755	33.27%

## Abbreviations

ACT: Artemisinin-based combination therapies
E.C.: Ethiopian calendar
G.C.: Gregorian calendar
HC: Health center
HEP: Health Extension Program
HSDP: Health Sector Development Plan
IRS: Insecticide residual sprays
ITN: Insecticide-treated bed nets
LLINs: Long-lasting insecticide-treated bed nets
MIS: Malaria Indicator Survey
NSP: National Strategic Plan
P. f.: * Plasmodium falciparum*
P. v.: * Plasmodium vivax*
RBM: Roll Back Malaria
SNNPR: Southern Nations Nationalities and Peoples Region
SPR: Slide positivity rate
SUFI: Scale-up of malaria interventions for impact
UN: United Nations
US: United States
US$: US dollars
WHA: World Health Assembly
WHO: World Health Organization
WSU: Wolaita Sodo University
WZHD: Wolaita Zone Health Department.
